# Infections associated with CAR‐T cell therapy in patients with relapsed refractory multiple myeloma: Risks and prevention strategies

**DOI:** 10.1002/cam4.7372

**Published:** 2024-06-24

**Authors:** Jing An, Jie Zhao, Ping Zou, Yicheng Zhang, Junni Wei, Weiwei Tian, Jia Wei

**Affiliations:** ^1^ Department of Hematology, Shanxi Bethune Hospital, Shanxi Academy of Medical Sciences Third Hospital of Shanxi Medical University, Tongji Shanxi Hospital Taiyuan Shanxi China; ^2^ School of Public Health Shanxi Medical University Taiyuan Shanxi China; ^3^ Sino‐German Joint Oncological Research Laboratory Shanxi Bethune Hospital, Shanxi Academy of Medical Sciences Taiyuan Shanxi China; ^4^ Institute of Hematology, Union Hospital, Tongji Medical College, Huazhong University of Science and Technology Wuhan China; ^5^ Department of Hematology, Tongji Hospital, Tongji Medical College Huazhong University of Science and Technology Wuhan Hubei China; ^6^ Immunotherapy Research Center for Hematologic Diseases of Hubei Province Wuhan Hubei China

**Keywords:** chimeric antigen receptor, infection, multiple myeloma, prevention strategy

## Abstract

**Background:**

Chimeric antigen receptor T (CAR‐T) cell therapy has emerged as a potent treatment for relapsed or refractory multiple myeloma, demonstrating significant clinical efficacy. Despite these advances, treatment‐related toxicities, particularly infections, pose a significant challenge to patient safety.

**Methods:**

This review synthesizes current knowledge on the mechanisms underlying post‐CAR‐T therapy infections, focusing on the interplay between immune dysfunction, host factors, and treatment‐induced toxicity. It provides a comprehensive analysis of the temporal and individual variability in infection characteristics and the confounding clinical presentation of cytokine release syndrome.

**Results:**

The review identifies that patients receiving CAR‐T cells are at increased risk of concurrent infections due to the heterogeneity in infection characteristics across different time periods, individuals, and patient groups. It highlights the diagnostic and therapeutic complexities introduced by the overlapping symptoms of infection and cytokine release syndrome.

**Conclusion:**

To enhance the infection control post‐CAR‐T therapy, this review proposes preventive strategies tailored to the early and long‐term management of patients. It underscores the need for a nuanced understanding of infection mechanisms and the importance of personalized prevention plans to improve clinical outcomes in multiple myeloma treatment.

## Graphical abstract

This review examines the significant clinical efficacy of chimeric antigen receptor T (CAR‐T) cell therapy in the treatment of relapsed or refractory multiple myeloma (RRMM), with a focus on the associated risks of infection and the development of preventive strategies. It elucidates the mechanisms of infections post‐CAR‐T therapy, summarizes the types and characteristics of infections during early monitoring and long‐term follow‐up, and proposes corresponding preventive measures to enhance the safety and efficacy of CAR‐T treatment for RRMM patients.

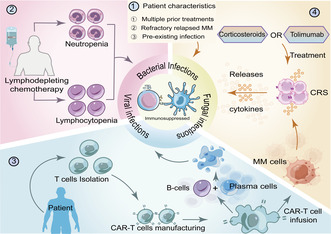

## 1 INTRODUCTION

Multiple myeloma (MM) is a common malignant tumor in clinical practice.^1^ Over recent decades, the survival of patients with MM has significantly improved with the widespread use of anti‐MM agents.^2^ Chimeric antigen receptor (CAR) T‐cell therapy has also provided a new regimen for the treatment of relapsed refractory MM (RRMM), showing substantial efficacy with controllable adverse reactions.^3^, ^4^, ^5^ The US Food and Drug Administration has approved the CAR‐T cell products idecabtagene vicleucel (ide‐cel) and ciltacabtagene autoleucel, which target the B‐cell maturation antigen (BCMA), for the treatment of RRMM. The CAR‐T product CT103A (IASO Bio), also known as IBI326 (Innovent), has also been approved for marketing in China. In addition to BCMA, other CAR‐T targets in RRMM currently in clinical research include the cluster of antigens 19 (CD19), 38 (CD38), and 138 (CD138), and G protein‐coupled receptor class C group 5 member D (GPRC5D) **(**Table 1 **)**.

**TABLE 1:** Effects of different CAR‐T targets for the treatment of relapsed refractory multiple myeloma on the immune system.

Targets	Expression of non‐malignant tumor cells	Multiple myeloma cell expression	Effect on the immune system
BCMA	Plasma cells, a few mature B cells	MM cell surface	Prolonged neutropenia and hypogammaglobulinemia due to depletion of normal plasma cells and B cells
GRPC5D	Limited to plasma cells	Several myeloma cell lines and in bone marrow plasma cells from patients with multiple myeloma	Prolonged neutropenia and hypogammaglobulinemia due to depletion of normal plasma cells
CD38	Progenitor B cells, plasma cells, T cells cells, NK cells, myeloid progenitor cells	Highly homogeneous expression in MM	Continuous depletion of B cells, plasma cells, leading to immune system dysfunction and hypogammaglobulinemia; action on T cells may lead to long‐term neutropenia
CD138	Plasma cells	MM cell surface	Prolonged neutropenia and hypogammaglobulinemia due to depletion of normal plasma cells
CD19	B cells	Expressed only in a small proportion of myeloma cells	Expressed only in B cells, predisposes to hypogammaglobulinemia

Abbreviations: BCMA, B‐cell maturation antigen; CD19, cluster of differentiation 19; CD38, cluster of differentiation 38, CD138, cluster of differentiation 138; GPRC5D, G protein‐coupled receptor family C 5 member D; MM, multiple myeloma.

Progress of research on CAR‐T cell therapy has been rapid. However, it is prone to various complications, with infection being one of the most common and an important cause of increased mortality.^6^ This is possibly due to patient factors but is also related to the treatment itself. Currently, patients with MM receiving CAR‐T cell therapy are mainly relapsed and refractory. These patients have the characteristics of receiving multiple lines of treatment, having a heavy tumor burden, an insufficient reserve of function of multiple organs, and susceptibility to immunosuppression.^6^ Lymphodepletion (LD) chemotherapy (usually fludarabine + cyclophosphamide) is often administered before CAR‐T cell infusion, resulting almost universally in early neutropenia and prolonged lymphopenia.^7^ In addition, after CAR‐T cell therapy, some patients have persistent neutropenia and lymphopenia due to autoimmune dysfunction and further destruction of CAR‐T cells, resulting in increased post‐treatment infection.^8^ The toxic side effects of CAR‐T cell immunotherapy can also lead to a sustained immunosuppressive state of CAR‐T cells.^9^, ^10^

Infection‐specific toxicities are currently poorly understood. In addition, most anti‐infective measures are derived from those used in other blood therapies, such as autologous or allogeneic bone marrow transplantation (hematopoietic cell transplantation [HCT]). Therefore, our aim was to examine the worldwide characteristics of CAR‐T cell infection in patients with RRMM, elucidate their risk factors, and propose corresponding prevention and treatment measures to provide a scientific basis for improving clinical efficacy in patients with RRMM.

## 2 MECHANISMS OF INFECTION ASSOCIATED WITH CAR‐T CELL THERAPY

**FIGURE 1:**
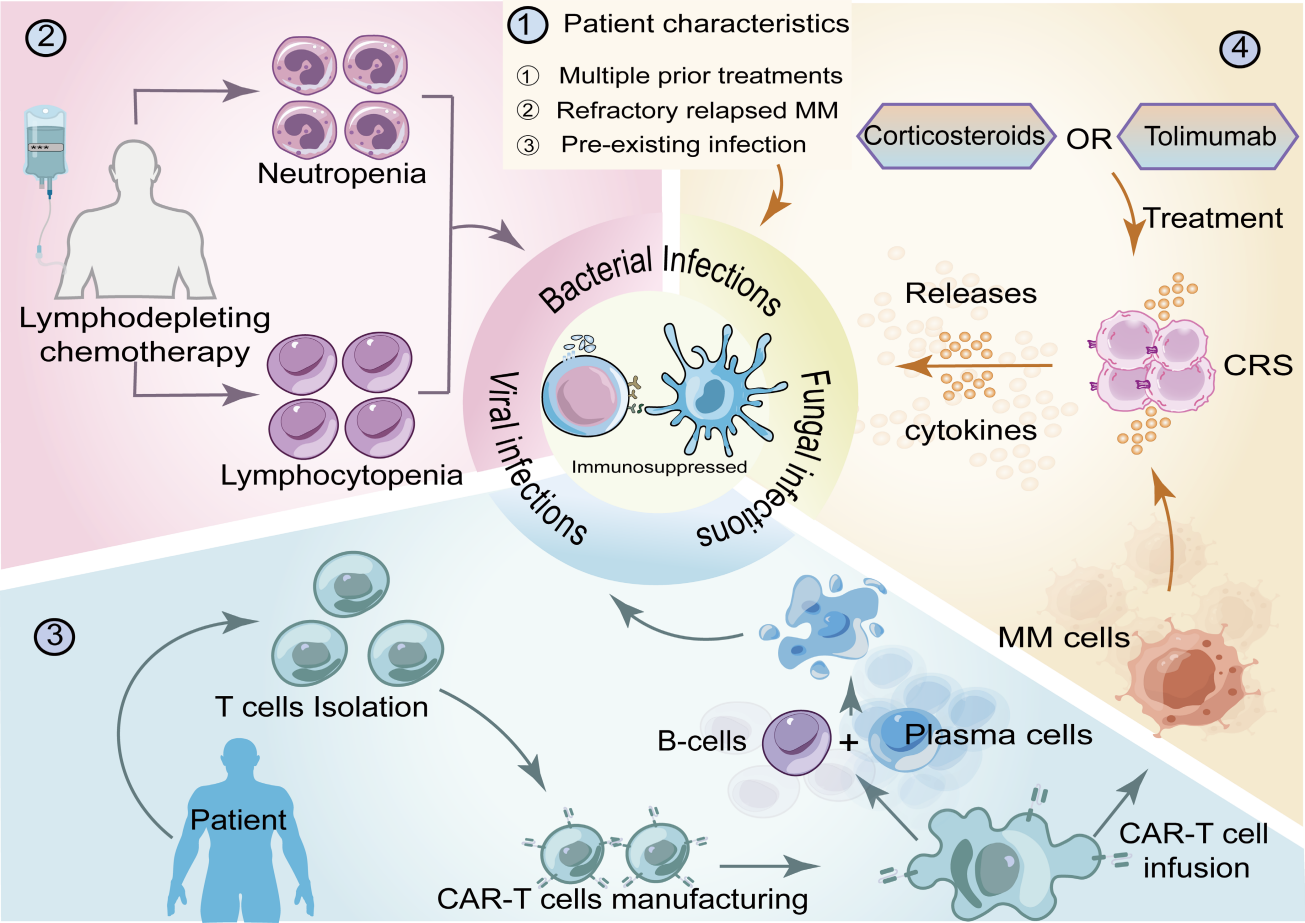
Mechanism of infection after CAR‐T. There are several factors that contribute to impaired immune function in patients receiving CAR‐T cell therapy, putting them at higher risk of infection after CAR‐T treatment. (1) Patients with multiple prior treatments, relapsed refractory multiple myeloma itself, and its prior infections. (2) Lymphodepletion prior to CAR‐T infusion resulted in loss of mucosal integrity and decreased neutrophils and lymphocytes. (3) The “on‐target to off‐tumor” effect continues to deplete plasma cells. (4) Treatment of CRS with the IL‐6 inhibitor tocilizumab and high‐dose corticosteroids after CAR‐T cell therapy further suppresses immune system function. At the same time, high levels of cytokines can suppress the patient's immune response, leading to the risk of infection. CAR, chimeric antigen receptor; CRS, cytokine release syndrome; MM, multiple myeloma.

Patients with RRMM treated with CAR‐T cells undergo complicated processes that lead to impaired immune function and, thus, infection^11^ **(**Figure 1 **)**.

First, MM inherently leads to immune dysfunction. Patients have received many different types of previous treatment, resulting in cumulative immune dysfunction.^12^, ^13^ The patient's underlying disease and previous antitumor regimens are established potential risk factors for infection.^14^ Preexisting latent infections may become established after CAR‐T treatment and reemerge if the immune system is compromised.

Secondly, another risk factor for infection is LD chemotherapy before CAR‐T infusion. Regulatory T cells and other immune cells must be cleared before CAR‐T infusion to enhance the function of T cells, improving the efficacy of CAR‐T cells against metastases.^15^ To date, fludarabine and cyclophosphamide have been used in combination. The resulting mucosal destruction and neutropenia may cause long‐lasting toxic side effects, with purine analogs such as fludarabine, potentially leading to infection.^16^, ^17^

Thirdly, using immunosuppressive therapy to treat the complications of CAR‐T cell therapy increases the risk of infection.^11^ Cytokine release syndrome (CRS) caused by CAR‐T cell activation, which usually presents with hyperthermia, hypotension, hypoxia, and organ toxicity, is one of the major adverse effects of CAR‐T cell therapy.^18^ Many cytokines can suppress the patient's immune response and increase their risk of infection. CRS is usually treated with the interleukin (IL)‐6 inhibitor tolimumab and high‐dose corticosteroids.^19^ These drugs may theoretically further suppress immune system function, leading to an increased incidence of infection.^20^

Fourthly, multiple RRMM targets of CAR‐T cells are expressed on both MM and normal B and plasma cells. This “on target to off tumor” effect continuously destroys normal plasma cells and some B cells in the human body, leading to an inevitable long‐term immune dysfunction and hypogammaglobulinemia after receiving CAR‐T cell therapy, making patients susceptible to infection.^21^

Finally, long‐term neutropenia is another important factor. It has been reported that, after CAR‐T cell therapy, neutropenia is associated with the LD chemotherapy received in the early phase, whereas in the late phase it is predominantly a cytokine‐mediated neutropenia.^11^ During this time, the patient's immunity significantly decreases, and their infection rate increases.

## 3 INCIDENCE AND SPECTRUM OF INFECTION AFTER CAR‐T CELL THERAPY

### 3.1 Infection rates

**FIGURE 2:**
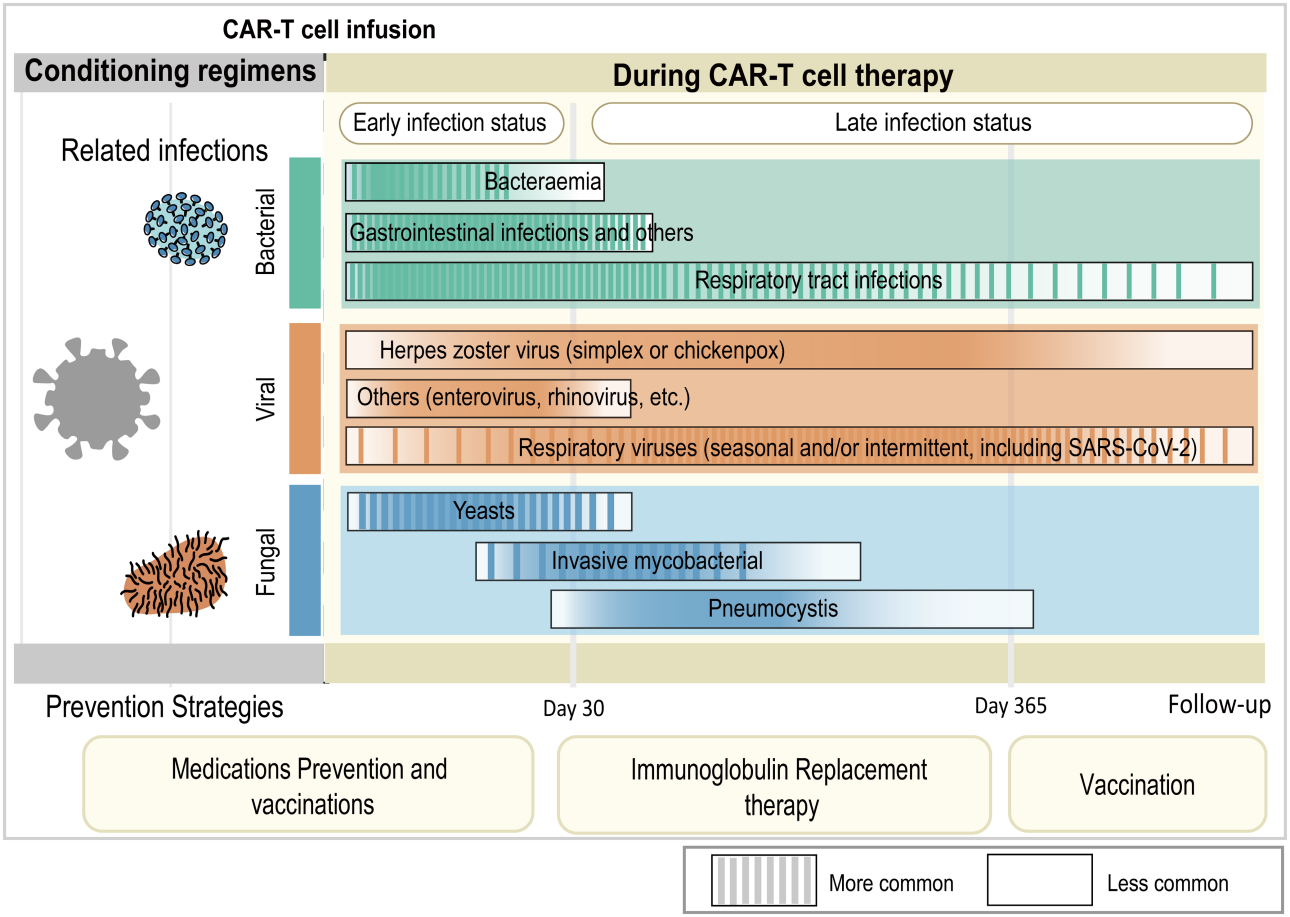
Infections occurring at different times following chimeric antigen receptor T‐cell therapy and strategies for prevention. Infections that developed within 30 days  following conditioning and CAR‐T infusion were called early infections, and those that developed 30 days to 365 days after CAR‐T infusion and later were called late infections. The types of infections vary across periods. Bacterial infection is the main type of early infection, and respiratory bacterial infection is the most frequent; late infection is the main viral infection; although fungal infection is rare, it is still occasionally occurs in the early stage. CAR, chimeric antigen receptor.

CAR‐T cell therapy can be divided into three periods: the LD chemotherapy regimen period, 0–30 days, and 30 to ≥365 days. The type of infection at each stage varies with time **(**Figure 2 **)**. Bacterial infections are prevalent early in CAR‐T cell therapy, with one‐third of early infections caused by bacteria.^22^ Previous reports have shown early bacterial infection rates of between 18.8% and 34.6% after CAR‐T cell therapy.^22^, ^23^, ^24^ A large proportion of severe infections are bacterial in origin, with 4.2%–18.8% being severe or life‐threatening.^23^, ^24^ Viruses and fungi show lower early infection rates than bacteria, with 12.5%–15.4% of early viral infections^22^, ^24^ and 3.8% of early fungal infections.^22^ Late infections are increasingly dominated by viral infections.^25^, ^26^, ^27^, ^28^, ^29^, ^30^, ^31^, ^32^, ^33^, ^34^, ^35^, ^36^, ^37^ After Day 30, viral infections account for 18.7%–35.7% of all infections, with mild and moderate viral infections being more common.^32^, ^37^ While later viral infections are more common than bacterial infections, the incidence of bacterial infections remains high, with late bacterial infection rates ranging from 14.9% to 57.5%.^26^, ^27^, ^28^, ^29^, ^37^ Late fungal infection rates range from 2.2% to 11.9%.^27^, ^28^, ^29^, ^37^

**FIGURE 3:**
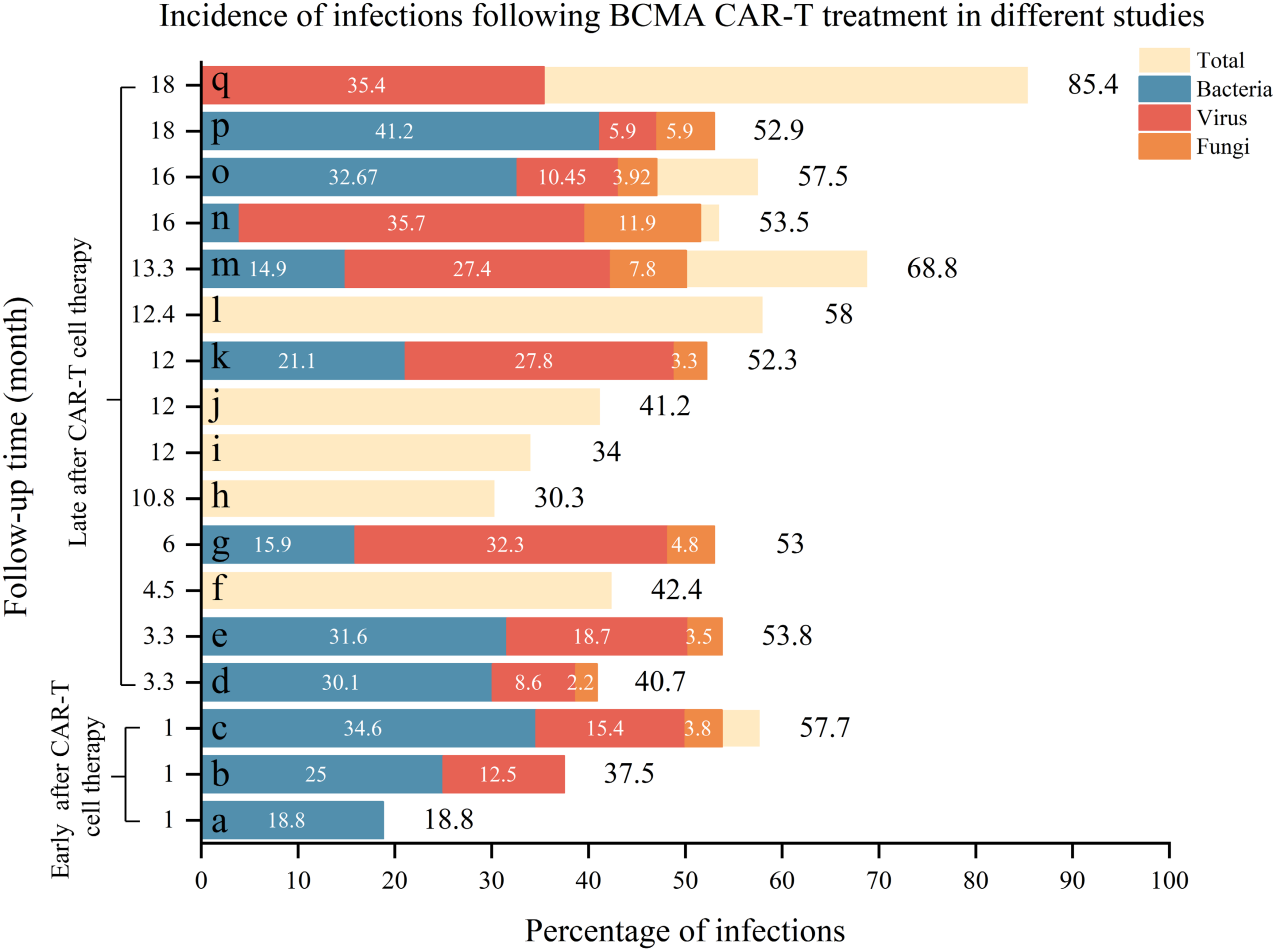
Incidence and frequency of infections after BCMA CAR‐T treatment by time point and type of infection. a (Luo et al.^23^), b (Mikkilineni et al.^24^), c (Mikkilineni et al.^22^), d (Little et al.^30^), e (Logue et al.^32^), f (Raje et al.^28^), g (Josyula et al.^35^), h (Zhou et al.^34^), i (Hansen et al.^33^), j (Cornell et al.^31^), k (Kambhampat et al.^25^), l (Berdeja et al.^29^), m (Munshi et al.^48^), n (Mailankody et al.^37^), o (Wang et al.^26^), p (Xu et al.^27^), q (Mi et al.^36^). BCMA, B‐cell maturation antigen; CAR, chimeric antigen receptor.

Existing studies of CAR‐T cell therapy in patients with MM have mainly focused on BCMA targets, with a few studies exploring other targets. Tables 2 and 3 summarize the infection characteristics of patients with RRMM after BCMA CAR‐T cell therapy.^25^, ^26^, ^27^, ^28^, ^29^, ^30^, ^31^, ^32^, ^33^, ^34^, ^35^, ^36^, ^37^ In single‐target (BCMA) CAR‐T cell therapy, the infection rates and types reported by different studies have varied between 30 and 540 days after treatment **(**Figure 3 **)**. Two studies examined patients with RRMM receiving ide‐cel therapy reported that the infection rate after ide‐cel therapy was 68.8% after 13 months and 34.0% after 1 year.^33^, ^38^ Besides BCMA‐targeted CAR‐T cell therapy, Mailankody et al.^39^ reported that three of 17 patients (17.6%) treated with GPRC5D‐targeted CAR‐T cell therapy (MCARH109) developed an infection within the first year after treatment.

**TABLE 2:** Data on infection complications in patients with relapsed refractory multiple myeloma treated with BCMA CAR‐T cells in prospective clinical trials.

	Mikkilineni et al.^22^	Xu et al.^27^	Raje et al.^28^	Mikkilineni et al.^24^	Wang et al.^26^	Munshiet al.^48^	Little et al.^30^	Cornell et al.^31^	Berdeja et al.^29^	Mi et al.^36^	Mailankody et al.^37^
Number of patients	26	17	33	24	40	128	27	17	97	48	52
Median age	56	56	60	55	55	61	59	56	61	61	64
Median prior lines of treatment	NR	4 (3–11)	7 (3–23)	3–5, >5	4 (2–9)	6 (3–16)	NR	5.5 (3–8)	6 (4–8)	4 (3–9)	5 (3–11)
Allo HCT, *n* (%)	0 (0)	0 (0)	0 (0)	0 (0)	NR	0 (0)	0 (0)	NR	8 (8.2)	0 (0)	NR
Auto HCT, *n* (%)	22 (84.6)	8 (47.1)	32 (97)	20 (83.3)	NR	0 (0)	20 (74.1)	NR	87 (89.7)	17 (35.4)	NR
Observation time	0‐30d	18 m	6.2–22.8 m	0‐30d	16 m	13.3 m	0–3.3 m	12 m	12.4 m	18 m	16 m
Number of infections	15 events in 11 pt	9 events in 9 pt	14 events in 14 pt	13 events in 9 pt	44 events in 23 pt	157 events in 88 pt	19 events in 11 pt	7 pt	57 events in 56 pt	48 events in 41 pt	27 events in 23 pt
Degree of infection, *n* (%)											
Mild to moderate	NR	NR	2 (14.3)	NR	0 (0)	28 (31.8)	4 (21.1)	NR	NR	NR	NR
Severe and life‐threatening	1 (9.1)	NR	0 (0)	NR	22 (50.0)	NR	15 (78.9)	NR	19 (33.3)	15 (37.5)	10 (23.3)
Death	NR	NR	0 (0)	1 (11.1)	0 (0)	NR	0 (0)	NR	1 (1.8)	NR	NR
Infection site, *n* (%)											
Respiratory tract	NR	7 (77.7)	5 (35.7)	NR	32 (72.7)	NR	2 (10.5)	3 (21.0)	23 (40.4)	33 (68.7)	4 (9.3)
Bloodstream	NR	0 (0)	NR	NR	4 (9.1)	NR	6 (31.6)	NR	4 (7.0)	0 (0)	NR
Intestinal tract	NR	0 (0)	NR	NR	4 (9.1)	NR	NR	NR	NR	NR	NR
Others	NR	2 (22.2)	2 (14.3)	NR	4 (9.1)	NR	2 (10.5)	NR	NR	NR	NR

Abbreviations: CAR, chimeric antigen receptor; G‐CSF, granulocyte colony‐stimulating factor; HCT, hematopoietic cell transplant; IVIG, intravenous immunoglobulin; NR, not reported; PT, patient; TCZ, tocilizumab.

**TABLE 3:** Data on infection complications in patients with relapsed refractory multiple myeloma treated with BCMA CAR‐T cells in retrospective studies.

	Luo et al.^23^	Kambhampati et al.^25^	Logue et al.^32^	Hansen et al.^33^	Zhou et al.^34^	Josyula et al.^35^
Baseline characteristics						
Number of patients	16	55	52	196	33	32
Median age	55	62	66	64	58	64
Median prior lines of treatment(range)	>6	6 (1–13)	6 (4–13)	7 (4–19)	4 (2–7)	8 (4–18)
Allo HCT, *n* (%)	0 (0)	1 (1.8)	0 (0)	0 (0)	0 (0)	6 (18.8)
Auto HCT, *n* (%)	7 (43.8)	48 (87.3)	42 (80.8)	164 (83.7)	7 (30.4)	26 (81.2)
Observation time	0‐30d	0‐12 m	0–3.3 m	0‐12 m	10.8 m	0‐6 m
Number of infections	3 events in 3 pt	47 events in 29 pt	46 events in 28 pt	67 pt	12 events in 10 pt	23 events in 17 pt
Degree of infection, *n* (%)						
Mild to moderate	1 (6.3)	43 (91.5)	14 (30.4)	NR	NR	17 (73.9)
Severe and life‐threatening	2 (12.5)	4 (6.4)	NR	NR	NR	6 (26)
Death	0 (0)	0 (0)	0 (0)	NR	NR	0 (0)
Infection site, *n* (%)						
Respiratory tract	2 (12.5)	32 (68.0)	11 (23.9)	NR	NR	NR
Bloodstream	1 (6.3)	1 (2.0)	2 (4.3)	NR	NR	3 (17.6)
Intestinal tract	0 (0)	0 (0)	6 (13.0)	NR	NR	NR
Others	0 (0)	14 (30.0)	28 (60.9)	NR	NR	NR
TCZ	NR	42 (76.4)	44 (84.6)	113 (57.7)	15 (45.5)	2 (6.2)

Abbreviations: CAR, chimeric antigen receptor; G‐CSF, granulocyte colony‐stimulating factor; HCT, hematopoietic cell transplant; IVIG, intravenous immunoglobulin; NR, not reported; PT, patient; TCZ, tocilizumab.

Infection rates have varied widely in most studies due to differences in patient characteristics, CAR‐T cell‐related factors, and chemoprophylaxis. For example, Luohui et al.^23^ reported a marked difference from Mikkilineni et al.^22^ in infection rates (18.8% vs. 57.7%) over the same follow‐up time (30 days). Luohui et al. examined a small number of patients (*n* = 16) with RRMM, all treated with traditional antimicrobial precautions.^23^ Mikkilineni et al. included 26 patients with RRMM, most of whom had ≥3 lines of previous therapy before CAR‐T cell therapy, with the number of previous therapies correlating positively with infection risk (*P* = 0.0064),^22^ and possibly accounting for the higher infection rate. In addition, patients receiving fewer lines of therapy before treatment have shown faster recovery of B‐cell counts, accompanied by a lowering their infection rate.^26^

Recently, some relevant studies have examined dual‐target CAR‐T cell therapy in patients with RRMM (Table 4). Whether CAR‐T cells binding to multiple targets leads to higher infection rates than their binding to a single target remains controversial and inconsistent. One study has shown that the 3‐month post‐treatment infection rate was 45% when cotargeting BCMA and CD19, which was similar to the infection rate with monotherapy.^40^ In another study, 12 (30.3%) patients treated with BCMA‐targeted CAR‐T cell monotherapy and 16 (45.7%) patients treated with BCMA and CD19 CAR‐T cell co‐therapy developed infections during a mean follow‐up time of 20.5 months. In the first 18 months, Cox model analysis did not show a significant difference in infection rates between these two groups. However, the cumulative infection rate was significantly higher in the CAR‐T cell co‐therapy group (45.5% vs. 31.7%), probably because the anti‐CD19 therapy prolongs the recovery time of humoral immunity, increasing the risk of long‐term infection.^34^

In addition, two studies have examined BCMA and CD38 CAR‐T cell co‐therapy in patients with RRMM, reporting that 37.5%^41^ and 21.7^42^ of patients developed an infection within 2 months. These studies did not show an increase in infection rates compared to patients receiving BCMA‐targeted CAR‐T cell therapy alone. However, the results are limited by the small number of patients included and the low CAR‐T cell infusion dose.^42^ In addition, other studies have examined dual GPRC5D and CD3 targeting in patients with RRMM. Infection rates varied by infusion dose, with 30 patients receiving a weekly dose of 405 μg/kg, of whom 37% developed infections (with one developing a Grade 3 COVID‐19 pneumonia), and 23 patients receiving a biweekly dose of 800 μg/kg, of whom 13% developed infections (with one developing a Grade 3 pneumonia septicemia).^43^ Therefore, whether dual‐targeting has a higher infection rate than single‐targeting needs further validation in prospective studies .

**TABLE 4:** Infection characteristics of dual‐target CAR‐T in treatment of RRMM.

	Krishnan et al.^43^		Mei et al.^41^	Tang et al.^42^	Wang et al.^40^	Zhou et al.^34^
Number of patients	30	23	16	23	62	35
Median age (range)	61.5 (46–80)	60.0 (47–84)	58.5 (48–78)	59 (49–72)	58 (30–69)	57 (45–66)
Median prior lines of treatment (range)	NR	NR	3 (2–3)	4 (2–9)	4 (2–17)	4 (2–5)
Lymphodepletion, *n* (%)	NR	NR	16 (100.0)	23 (100.0)	62 (100.0)	35 (100.0)
Auto HCT, *n* (%)	NR	NR	3 (18.8)	3 (13.0)	17 (27.0)	7 (28.0)
Other targets	GPRC5D + CD3		BCMA + CD38	BCMA + CD38	BCMA + CD19	BCMA + CD19
CAR‐T cell dose	405 μg/kg	800 μg/kg	2.1 × 10^6^/kg	4.0 × 10^6^/kg	NR	NR
Observation time (month)	7.5 (0.9–15.2)	3.7 (0.0–12.0)	0–2	NR	0–3	20.5 (12–27)
Infection rate, *n* (%)	37	13	6 (37.5)	5 (21.7)	28 (45.2)	16 (45.7)
Tocilizumab, *n* (%)	NR	NR	NR	4 (17.4)	20 (32.3)	10 (28.6)
Corticosteroids, *n* (%)	NR	NR	3 (18.8)	3 (13.0)	23 (37.1)	NR

Abbreviations: CAR, chimeric antigen receptor; HCT, hematopoietic cell transplant; NR, not reported.

### 3.2 Infection type

#### 3.2.1 Bacterial infections

Neutropenia is the most significant risk factor for bacterial infections. In addition, many patients have received antimicrobial therapy and multiple chemotherapies before CAR‐T cell therapy, severely disrupting the composition of their microbiome.^24^, ^25^, ^38^, ^44^, ^45^, ^46^ Early bacterial infections usually occur within the first 2 weeks of neutropenia and manifest as bacteremia or organ‐specific infections. Different studies have shown that the most common sites of infection are the pulmonary system, respiratory system, blood, and skin or soft tissues, with the most common bacteria types being Gram‐negative bacteria and maltophilic narrow‐feeding *Aeromonas*.^25^, ^32^ Other less common bacteria include *Klebsiella pneumoniae*, coagulase‐negative *Staphylococci*, *Mycobacterium tuberculosis*, *Clostridium difficile*, *Escherichia coli*, and *Acinetobacter baumannii*.^32^, ^47^ Patients with bacterial infections who are heavily exposed to broad‐spectrum antibiotics are more susceptible to multidrug‐resistant bacterial infections in the hospital, increasing their risk of multidrug‐resistant bacterial colonization and invasive infections during the neutropenic phase.^38^

#### 3.2.2 Viral infections

Viral infections are mainly attributable to the fact that patients receive lymphocyte chemotherapy before treatment and therefore have a severe hypogammaglobulinemia.^48^ Lymphopenia (B or T lymphocytes) and hypogammaglobulinemia have emerged as two critical components of immune dysfunction after ≥30 days of treatment.^44^ B‐cell aplasia and hypogammaglobulinemia increased the infection risk and predisposed patients to persistent infection.^26^ One study reported that patients had high rates of hypogammaglobulinemia (up to 98%) within 3 months of treatment. Lymphopenia persisted for up to 1 year after CAR‐T cell therapy, and the prevalence of lymphopenia remained at 84% after 1 year. Therefore, this study suggests that viral infection may be associated with greater lymphopenia and hypogammaglobulinemia in these patients after CAR‐T cell therapy.^25^ Individuals presenting with severe lymphocytopenia and hypogammaglobulinemia are predisposed to a heightened susceptibility to infections from respiratory syncytial virus (RSV) and other pathogens, such as varicella‐zoster virus (VZV), cytomegalovirus (CMV), and severe acute respiratory syndrome coronavirus 2 (SARS‐CoV‐2). As a result, the prevalence of RSV, VZV, and SARS‐CoV‐2 infections is notably higher compared to those caused by less frequently encountered viruses, including polyomaviruses, rhinoviruses, and enteroviruses.^32^ In recent years, SARS‐CoV‐2 infections have occasionally led to patient death after BCMA‐targeted CAR‐T cell therapy. Previous studies have shown an increased risk of severe COVID‐19 and adverse outcomes in patients treated with CAR‐T cell therapy, with lymphopenia being an independent risk factor for COVID‐19 severity.^32^, ^49^ Spanjaart et al.^50^ reported an attributable mortality of 41% in patients diagnosed with COVID‐19 after BCMA CAR‐T cell therapy. However, as the virus mutates, the fatality rate decreases. For example, a recent study showed that SARS‐CoV‐2 attributable mortality in adult CAR‐T cell therapy recipients was 4.3%.^51^

#### 3.2.3 Fungal infection

Fungal infections are less common in patients with RRMM receiving CAR‐T cell therapy. Among them, the most important risk factors in patients with immune effector cell‐associated neurotoxicity syndrome (ICANS) are the course of neutropenia (and, in some cases, lymphopenia), severe CRS, and long‐term systemic corticosteroid use. The most frequently reported cases were severe yeast and invasive fungal infections.^25^, ^52^ In a study of BCMA‐targeted CAR‐T cell therapy, two patients with fungal infections developed mycotic infections, one of whom had persistent neutropenia at the time of infection.^25^ Studies have rarely demonstrated *Pneumocystis carinii* infections, probably reflecting the effectiveness of routine preventive measures.^25^

## 4 RISK FACTORS FOR INFECTIOUS COMPLICATIONS

Risk factors for infection after CAR‐T cell therapy in patients with RRMM have been inconsistently reported. Several studies have shown that neutropenia (absolute neutrophil count [ANC] < 500 cells/mm^3^) and lymphopenia (absolute lymphocyte count <200 cells/mm^3^) after LD are risk factors for developing infections.^22^, ^24^, ^35^ In addition, an increased number of previous lines of therapy, previous infections, and a longer time from the last bridging therapy to LD are independent risk factors for developing infection.^24^, ^32^ However, Kambhampati et al.^25^ found no statistically significant risk factors for infection, despite an increased infection tendency with >3 previous lines of therapy, use of bridging chemotherapy use within 30 days before CAR‐T cell therapy, lymphopenia, and hypogammaglobulinemia after CAR‐T cell therapy. In addition, infection rates were higher in immunoglobulin G (IgG) than in non‐IgG patients with MM (0.50 vs. 0.31 person days/100).^35^

## 5 DIFFERENTIATING BETWEEN INFECTION AND CRS

Fever is a common symptom of CRS. In severe cases, CRS also presents with sepsis‐like symptoms, hypotension, hypoxia, multiple organ dysfunction, and an increase in the inflammatory cytokines IL‐6 and C‐reactive protein (CRP).^53^ This presentation poses significant diagnostic difficulties and complicates the management of infections. Current techniques used to diagnose infection are not sufficiently specific to distinguish between infections and CRS, making the clinician's empirical judgment particularly important. Most fevers caused by CRS occur earlier and are predominantly hyperthermic than those due to infections. However, peak fever will probably decrease after admission in most patients. CRS should be considered in the absence of clear evidence of infection during a fever and the presence of CAR T‐cell expansion and elevated levels of IL‐6 and other cytokines. However, clinicians must be alert to the possibility of new infections, which can occur at any time during CRS. Due to its high efficiency, broad spectrum, and low bias, metagenomic next‐generation sequencing is a suitable reference method in the early stages.^54^ Changes in certain specific cytokines may also help to distinguish between CRS and infections following CAR‐T. Shao et al.^55^ found that coagulation parameters and the levels of some cytokines (IL‐6, IL‐10, and interferon [IFN]‐γ) correlated positively with CRS severity. Patients with severe CRS are mainly characterized by elevated serum levels of angiopoietin 2 (ANGPT2) and von Willebrand factor (VWF), and these can also be used to distinguish CRS from infection.^56^ It is vital to distinguish CRS from infection since there are different treatments for these two pro‐inflammatory diseases. While both IL‐6 inhibitors and corticosteroids effectively reduce toxicity, antimicrobial therapy should be instituted early when infection develops. Additional large‐scale studies are needed to understand the differences between CRS and infection better.

## 6 STRATEGIES FOR PREVENTING INFECTION

With the approval and commercial release of multiple CAR‐T cell therapy products, alongside the emergence of large‐scale clinical research and innovative therapies, numerous authoritative medical institutions have formulated a series of recommendations for the prevention and control of infections following CAR‐T cell therapy, particularly for CD19‐targeted treatments. These guidelines, which draw heavily from the established practices of hematopoietic stem cell transplantation (HSCT), detail various infection risk categories, pathogen types, and preventive strategies.^57^, ^58^, ^59^ However, it is important to recognize that while these recommendations are grounded in historical experience and aimed at guiding clinical practice, they exhibit notable differences in perspectives. Such disparities underscore the multifaceted considerations required to formulate the most effective infection prevention protocols in actual clinical settings. Against this backdrop, the present article synthesizes existing data to provide practical preventive recommendations for infections in RRMM patients post‐CAR‐T cell therapy, encompassing pharmacological prophylaxis and vaccination strategies **(**Table 5 **)**.

**TABLE 5:** Infection prevention strategies following CAR‐T in RRMM.

Type of infection	Preventive strategy				
	Pharmacologic prophylaxis		Vaccination		
	Drugs	Conditions of Use	Vaccines	Inoculation conditions	
				Before CAR‐T	Post‐CAR‐T
Bacterial infection	Fluoroquinolones (levofloxacin, ciprofloxacin et al.)	Routine prophylaxis was not required during follow‐up and was used when ANC was less than 0.5 × 10^9^/L	Inactivated vaccines (pneumococcal, Haemophilus influenzae type b, diphtheria and pertussis vaccines, etc.)	–	Antibody titers were performed at 6 months, and if conditions were met: 3 doses of vaccine (2 doses 1–2 months apart) within 6–12 months: serum IgA and B‐cell counts >20/μL; CD4 + T cell counts >200/μL
	G‐CSF	Although controversial, it is recommended to consider use in patients with prolonged neutropenia			
Virus infection	Acyclovir, Valacyclovir et al.	For prevention of herpes virus family viruses, acyclovir 400–800 mg BD recommends continuous use for 1 year after CAR‐T	Influenza vaccine	2 weeks before lymphodepleting	>3 month vaccination
			SARS‐COV‐2	2 weeks before lymphodepleting	Re‐vaccinate 3–6 months with a full course of vaccine, with the first booster dose 3 months later and the second booster dose 6 months later
	Entecavir, Lavmidine et al.	HBs Ag positive or HBs Ag negative, anti‐HBc Ab IgG positive: received entecavir prophylaxis for ≥6 months	Inactivated vaccine	–	>3 months after CAR‐T
			Live/non‐live adjuvanted vaccines	–	>24 months after CAR‐T and complete immune reconstitution
Fungal infection	Posaconazole, fluconazole, voriconazole, or isavuconazole	Fluconazole 200–400 mg every 24 h Or consider posaconazole 300 mg Daily	–	–	–
	Co‐trimoxazole	PJP:Methicillin sulfamethoxazole 800/160 mg，duration for 6–12 months	–	–	–

Abbreviations: CAR, chimeric antigen receptor; G‐CSF, granulocyte colony‐stimulating factor; PJP, pneumocystis jiroveci pneumonia.

### 6.1 Prevention of bacterial infections

During the initial phase of CAR‐T cell therapy, the incidence of bacterial infections is high, with the majority of severe infection events originating from bacteria. Therefore, the selection of antimicrobial prophylaxis should be based on guideline recommendations, clinical practice, and the variability of local epidemiological data.^6^ Historical data indicate that neutropenia is a primary risk factor for bacterial infections, prompting clinicians to consider the duration of neutropenia.^59^ For patients with significant neutropenia (ANC <0.5 × 10^9^/L), antimicrobial prophylaxis is recommended and should continue until the neutrophil count recovers above a safe threshold.^57^, ^58^, ^59^ Levofloxacin is the preferred agent for preventing Gram‐negative bacterial infections, while ciprofloxacin and teicoplanin have also been studied for antimicrobial prophylaxis.^23^, ^24^ CAR‐T therapy should be delayed for patients with active and uncontrolled infections until the infection disappears, including LD chemotherapy and CAR‐T cell infusion.^58^

Beyond routine antimicrobial use, the role of granulocyte colony‐stimulating factor (G‐CSF) has garnered considerable attention. However, its specific function in CAR‐T cell therapy remains to be fully elucidated. Existing studies reveal a complex relationship between G‐CSF use and clinical outcomes post‐CAR‐T cell therapy. On one hand, G‐CSF may influence the efficacy of CAR‐T cells by activating myeloid‐related cytokines and potentially exacerbating the severity of CRS and ICANS.^60^, ^61^ On the other hand, some research suggests that G‐CSF may help shorten the duration of neutropenia post‐CAR‐T cell therapy, thereby reducing the risk of infection.^26^ Given these findings, the application strategy for G‐CSF in CAR‐T cell therapy requires additional clinical data and research to clarify its effects. Currently, G‐CSF is primarily recommended for patients experiencing prolonged neutropenia, to improve their clinical outcomes.^62^, ^63^ Future research should further explore the optimal timing and dosage of G‐CSF in CAR‐T cell therapy and its impact on overall treatment efficacy and safety.

### 6.2 Prevention of viral infections

Viral infections are a common complication during the CAR‐T cell therapy process for MM, with a significant increase in incidence beyond 30 days of treatment. Consequently, preventive measures against viral infections are crucial when administering CAR‐T therapy to RRMM patients. Compared to CD19 CAR‐T therapy for B‐cell lymphomas and acute B‐lymphoblastic leukemia, varicella‐zoster virus (VZV) infections appear more frequently in BCMA CAR‐T cell treatment for RRMM patients.^11^ In light of this, we recommend that all RRMM patients scheduled to receive BCMA CAR‐T cell therapy receive prophylactic treatment with acyclovir or valacyclovir during therapy until their immune system fully recovers, with a suggestion to continue for several months.^64^

As CAR‐T therapy becomes increasingly prevalent in clinical practice, expanding to patient populations including those with chronic infections, vigilance is required for the potential risk of viral reactivation, especially for hepatitis B virus (HBV) carriers.^62^ For HBsAg‐positive patients or those with a history of HBV infection (HBsAg negative and anti‐HBV core antibody [HBcAb] IgG positive) who have been on long‐term anti‐HBV medication (such as entecavir), prophylactic treatment with entecavir for at least 6 months is recommended to reduce the risk of viral reactivation, along with strict monitoring through liver function tests or HBV DNA testing.^65^ Additionally, studies have explored the antiviral prophylactic effects of entecavir and lamivudine in HBsAg‐positive patients, demonstrating effective viral control.^66^ Therefore, before initiating CAR‐T cell therapy, it is essential to ensure effective suppression of HBV DNA levels in HBsAg‐positive patients to enhance treatment safety and efficacy.^66^, ^67^, ^68^

In summary, current prevention strategies for viral infections in CAR‐T therapy are primarily based on empirical therapies rather than specific etiological evidence. To more effectively prevent viral infections in CAR‐T treatment, there is an urgent need for more large‐scale, multicenter studies to provide robust evidence to support and guide optimized antiviral prevention strategies.

### 6.3 Prevention of fungal infections

While fungal infections are uncommon in patients receiving CAR‐T cell therapy, prophylactic antifungal therapy should be considered in some high‐risk patients with fungal infections who develop chronic granulocytopenia or who take chronic systemic corticosteroids for adverse events related to CAR‐T cell therapy.^57^, ^69^ Prophylactic antifungal infection treatment with azoles (e.g., posaconazole, fluconazole, or voriconazole) and micafungin is recommended.^58^ Prophylactic antifungal therapy with oral fluconazole after CAR‐T cell therapy is recommended for patients with delayed hematologic recovery until granulocytes return to normal.^51^, ^70^ Given the universal risk of lymphopenia and the associated risk of Pneumocystis jiroveci pneumonia (PJP), it is recommended that all patients use *co‐trimoxazole* for PJP prophylaxis when tolerated.^21^, ^71^ In cases where patients exhibit an allergic reaction to co‐trimoxazole, an alternative prophylactic regimen comprising atovaquone, primaquine, and clindamycin may serve as an effective substitute for infection prevention.^64^

### 6.4 Vaccination

Given the immunological dysregulation that often occurs in RRMM patients pre‐ and post‐CAR‐T cell therapy, the formulation of vaccination strategies is particularly critical. Although robust data on the optimal practices for vaccination are currently lacking, guidelines from allogeneic hematopoietic cell transplantation (HCT) and professional associations can provide essential guidance for these patients' vaccination plans.^72^

Before devising individualized vaccination plans, a comprehensive immune assessment of the patient should be conducted, including a detailed infection history and current immune status. For patients who have not been vaccinated or have suboptimal immunity, priority should be given to vaccination against influenza, pneumococcal disease, herpes zoster, and COVID‐19 provided that disease control is stable.^64^

For bacterial infections, antibody titers should be assessed 6 months post‐CAR‐T cell therapy to evaluate the suitability for vaccination with pneumococcal conjugate vaccines, diphtheria/tetanus/acellular pertussis vaccines, and *Haemophilus influenzae* type b vaccines.^73^ It is recommended to administer three doses over 6 to 12 months post‐therapy, with each dose spaced 1 to 2 months apart. If patients do not respond to the vaccines, subsequent doses should be paused until clear evidence of immune reconstitution emerges.^44^

Furthermore, to prevent viral infections in RRMM patients undergoing CAR‐T therapy, a comprehensive vaccination approach is also recommended. Regardless of the degree of immune reconstitution, influenza vaccines should be administered 2 weeks before LD chemotherapy and more than 3 months post‐CAR‐T cell therapy to reduce the risk of influenza infection. In the post‐pandemic era of COVID‐19, SARS‐CoV‐2 vaccination is particularly crucial for CAR‐T cell therapy patients. Given the potential for weaker immune responses to SARS‐CoV‐2 vaccines in these patients, vaccination strategies need reassessment.^74^, ^75^ It is recommended to perform a COVID‐19 nucleic acid test on patients before implementing CAR‐T cell therapy; if positive, treatment should be delayed until the patient is asymptomatic and has two consecutive negative nucleic acid tests.^76^ For patients not infected with SARS‐CoV‐2, the initial SARS‐CoV‐2 vaccination should be completed at least 2 weeks before LD chemotherapy, with a booster dose administered 3 to 6 months post‐CAR‐T therapy.^76^

For other types of vaccines, administration should be timed within specific windows. Inactivated vaccines should be given 3 months post‐CAR‐T cell infusion, while live attenuated vaccines, due to potential risks, should be administered at least 24 months post‐CAR‐T cell infusion in the absence of immunosuppression.^77^ The immune response status of patients should be precisely understood by assessing pathogen‐specific IgG levels and immune response levels post‐vaccination to devise more rational vaccination plans.^44^

Despite this, information on the effectiveness of vaccination during CAR‐T cell therapy remains limited. Future research should delve into the safety and efficacy of vaccination during CAR‐T cell therapy to further optimize treatment strategies for RRMM patients and provide more precise guidance for clinical practice.

### 6.5 Immunoglobulin replacement therapy

In CAR‐T cell therapy, intravenous immunoglobulin (IVIg) is also used for infection prevention, but its application has not been extensively studied, particularly regarding its impact on overall IgG levels and potential improvement in survival outcomes in CAR‐T cell therapy patients.^71^, ^78^ Currently, available data primarily derive from hematological cancer patient populations receiving anti‐CD20 monoclonal antibody therapy or allogeneic hematopoietic stem cell transplantation (HSCT).^79^, ^80^, ^81^, ^82^ Recent research findings offer some insights but have not reached a consensus. The study by Kambhampati et al.^25^ showed that patients receiving IVIg during CAR‐T cell therapy did not exhibit a significant reduction in infection rates. Similarly, Wang et al.^26^ found that the impact of IVIg on detectable immunoglobulin levels during CAR‐T cell therapy was negligible. These findings suggest that IVIg may not be a key factor in improving clinical outcomes for CAR‐T cell therapy patients. However, most clinical guidelines and expert recommendations suggest that IVIg may be considered as a replacement therapy for patients with IgG levels below 400 mg/dL or within the range of 400–600 mg/dL with a history of recurrent infections.^57^, ^83^, ^84^

In summary, while IVIg has some applications in preventing and treating infections, its general applicability and necessity for all patients still require further research and validation.

## 7 SUMMARY AND OUTLOOK

Although CAR‐T immunotherapy has demonstrated significant efficacy in the treatment of hematological malignancies, particularly RRMM, the high incidence of post‐treatment infections remains a critical clinical challenge. Extensive research indicates that RRMM patients face infection risks post‐CAR‐T cell therapy, with incidence, types, and severity influenced by various individual factors and changing over time. Bacterial infections predominate in the early stages of treatment, while viral infections are more common in the later stages, albeit typically mild to moderate. Notably, SARS‐CoV‐2 infections in CAR‐T cell therapy patients may lead to adverse outcomes. In contrast, fungal infections occur relatively infrequently.

Given the frequent occurrence of infections, the implementation of antimicrobial and antiviral prevention measures, along with vaccination strategies, is essential for preventing and controlling infections in CAR‐T cell therapy patients. Current clinical practice emphasizes the importance of preventive strategies to reduce the occurrence of infection‐related complications and improve overall patient outcomes.

In conclusion, while the efficacy of CAR‐T cell immunotherapy is widely recognized, complications such as infections remain key determinants of survival and quality of life for RRMM patients. We need more prospective, multicenter studies to further validate and refine existing infection prevention strategies. This includes comparing infection risks associated with different CAR‐T cell therapy targets and exploring the infection risks of dual or multi‐target CAR‐T cell therapies. In terms of prevention strategies, more data are needed to guide clinicians in developing personalized prevention plans, including the selection of antimicrobial, antiviral, and antifungal agents, as well as vaccination strategies. Additionally, the role of COVID‐19 vaccination in CAR‐T cell therapy patients should be given special attention in the context of the global SARS‐CoV‐2 pandemic. The potential role of immunoglobulin replacement therapy in CAR‐T cell therapy also warrants further investigation. With a deeper understanding of the mechanisms of infections related to CAR‐T cell therapy, more effective prevention strategies can be developed to improve the overall prognosis of RRMM patients.

## AUTHOR CONTRIBUTIONS

**Jing An:** Conceptualization (equal); data curation (equal); visualization (lead); writing – original draft (lead). **Jie Zhao:** Conceptualization (equal); data curation (equal); writing – original draft (lead). **Ping Zou:** Writing – original draft (equal). **Yicheng Zhang:** Writing – original draft (equal). **Junni Wei:** Writing – original draft (supporting). **Weiwei Tian:** Conceptualization (equal); supervision (equal); writing – review and editing (equal). **Jia Wei:** Conceptualization (equal); supervision (equal); writing – review and editing (equal).

## FUNDING INFORMATION

National High Technology Research and Development Program of China, Grant/Award Number: 2021YFA1101500; National Natural Science Foundation of China, Grant/Award Number: 82070217 and 81873427; Fundamental Research Program of Shanxi Province, Grant/Award Number: 202303021211224; 2023 COVID‐19 Emergency Project of Shanxi Bethune Hospital, Grant/Award Number: 2023xg02; Shanxi Bethune Hospital National Natural Seed Cultivation Program, Grant/Award Number: 2023GZRZ29.

## CONFLICT OF INTEREST STATEMENT

The authors declare no conflicts of interest.

## Data Availability

Data sharing is not applicable to this article as no new data were created or analyzed in this study.
